# Type specific Real time PCR for detection of human herpes virus 6 in schizophrenia and bipolar patients: a case control study

**DOI:** 10.1186/s12888-015-0662-z

**Published:** 2015-11-20

**Authors:** Jila Yavarian, Somaye Shatizadeh Malekshahi, Roya Yavarian, Shaghayegh Yazdani, Leila Janani, Nazanin Zahra Shafiei Jandaghi, Seyed Jalal Kiani, HamidReza Ahamadkhaniha

**Affiliations:** 1 Department of Virology, School of Public Health, Tehran University of Medical Sciences, Tehran, Iran; 2 Department of Psychiatry, Medical School, Urmia University of Medical Sciences, Urmia, West Azarbayejan Iran; 3 Department of Biostatistics, School of Public Health, Iran University of Medical Sciences, Tehran, Iran; 4 Department of Psychiatry, Medical School, Iran University of Medical Sciences, Tehran, Iran

**Keywords:** Schizophrenia, Bipolar disorder, HHV-6

## Abstract

**Background:**

Schizophrenia (SC) and bipolar disorder (BD) are among the most devastating diseases worldwide. There are several lines of evidence suggesting that viruses may play significant roles in the etiology of these mental disorders. The aim of this study was the detection of HHV-6A/B in the peripheral blood mononuclear cells (PBMC) of SC and BD patients versus the healthy control (HC) subjects using a new method of type-specific Real time PCR analysis.

**Methods:**

A type-specific Real time PCR was performed for simultaneous detection and typing of HHV-6A/B in the PBMCs of 120 SC and BD patients and 75 HCs.

**Results:**

Only one case of HHV-6B out of 120 (0.8 %) SC and BD patients and two cases of HHV-6A (2.7 %) in 75 HCs were detected.

**Conclusions:**

The low levels of HHV-6 detection in PBMCs, severely limited the capacity of this study to investigate the association between the presence of HHV-6 and BD or SC in this population, thus no conclusions can be drawn in this regard. Meanwhile this study introduces a Real time PCR based method for type specific detection of HHV-6A/B in clinical samples.

## Background

Schizophrenia (SC) and bipolar disorder (BD) are debilitating mental illnesses distributed worldwide. Various factors have been implicated in the etiology and pathogenesis of these major mental disorders. There is controversial evidence to indicate that infectious organisms might have a role in affecting the etiologic pathways implicated in psychiatric diseases such as SC and BD [1]. Viral agents, due to their potential latency and neurotropism are considered as possible agents in the pathogenesis of many central nervous system (CNS) disorders [2]. Herpes viruses have been supposed as the key pathogenic factors in mental diseases [3]. The human herpes virus 6 (HHV-6) was first isolated in 1986 by Salahuddin and coworkers [4, 5]. The HHV-6 virus infects most infants under the age of 12 months and almost 100 % of the population in adulthood is seropositive for this virus [6]. Two species of the virus (A and B) have been identified [7] with 88–96 % nucleotide sequence homology [8]. Human herpes virus 6B is the cause of the common childhood disease, exanthema subitum [9, 10]. The pathologic characteristics of HHV-6A are less known but it appears to be more neurotropic. Nowadays the number of diseases associated with HHV-6 infection is increasing. HHV-6 may play a pathogenic role in neurologic disorders such as epilepsy, multiple sclerosis as well as skin diseases [11–13].

As HHV-6 is predominantly a T-cell tropic virus, the purpose of this study was the detection of HHV-6A/B in the peripheral blood mononuclear cells (PBMC) of SC and BD patients versus the healthy control (HC) subjects using a type specific Real time PCR analysis.

## Methods

### Patients

Blood samples were obtained from 120 BD and SC patients (60 BD and 60 SC) diagnosed according to DSM-IV diagnostic criteria using the Structured Clinical Interview for DSM-IV Axis I Disorders (SCID-1) [14]. Patients were hospitalized between March to September 2013 in Iran Mental Hospital located in Tehran. The medical history and demographic data were recorded on standard forms. Patients with substance abuse, mental retardation, epilepsy, history of head trauma or encephalitis and any other neurologic disorder were excluded. The study was approved by the ethics committee of Tehran University of Medical Sciences. All participants signed written informed consent after explaining the study procedures.

A population of 75 healthy individuals without a history of mental and neurological illnesses and hospital admission were selected as control subjects [15]. Geographic region, sex, age and socioeconomic status were matched between the case and control groups.

### Preparation of PBMCs

For the isolation of PBMCs, 10 ml of blood was drawn by venipuncture in the presence of anticoagulant. The peripheral blood mononuclear cells were separated using lymphocyte separation medium (Ficoll-paque™-plus) and stored in liquid nitrogen until use.

### DNA extraction

High Pure Viral Nucleic acid kit (Roche, Germany) was used for DNA extraction from PBMCs. Concentration of the extracted DNA was determined by measuring absorbance at 260 nm.

### Beta-globin PCR

Beta-globin PCR with specific primers (Forward- ACACAACTGTGTTCACTAGC & Reverse- CAACTTCATCCACGTTCACC) was used as indicator of the quality of the extracted DNA from the blood samples.

### Type specific Real time PCR

We performed a Quantitative Real time PCR assay which can detect HHV-6A/B within one reaction by two probes that have only one nucleotide difference with different reporters and quenchers. The FAM/TAMRA probe detects HHV-6A and Joe/BHQ-1 probe detects HHV-6B. The sequence of the primers and probes are shown in Table 1.

**Table 1:** The sequences of type-specific Real time PCR primers and probes

Primers	Sequences
HHV6U41-F4	5′- CGGAACATTGTTGAGCAGAAA-3′
HHV6U41-R106	5′- AAGAAGAATCCCTTGTCTGGC-3′
Probes	Sequences
HHV6U41-ProbeA	FAM-CTCTAAGCACGAAATCTTCACATTCGGAAACA-TAMRA
HHV6U41-ProbeB	JOE-CTCTAAGCACGAAATTTTGACATTCGGAAACA-BHQ1

**Fig. 1:**
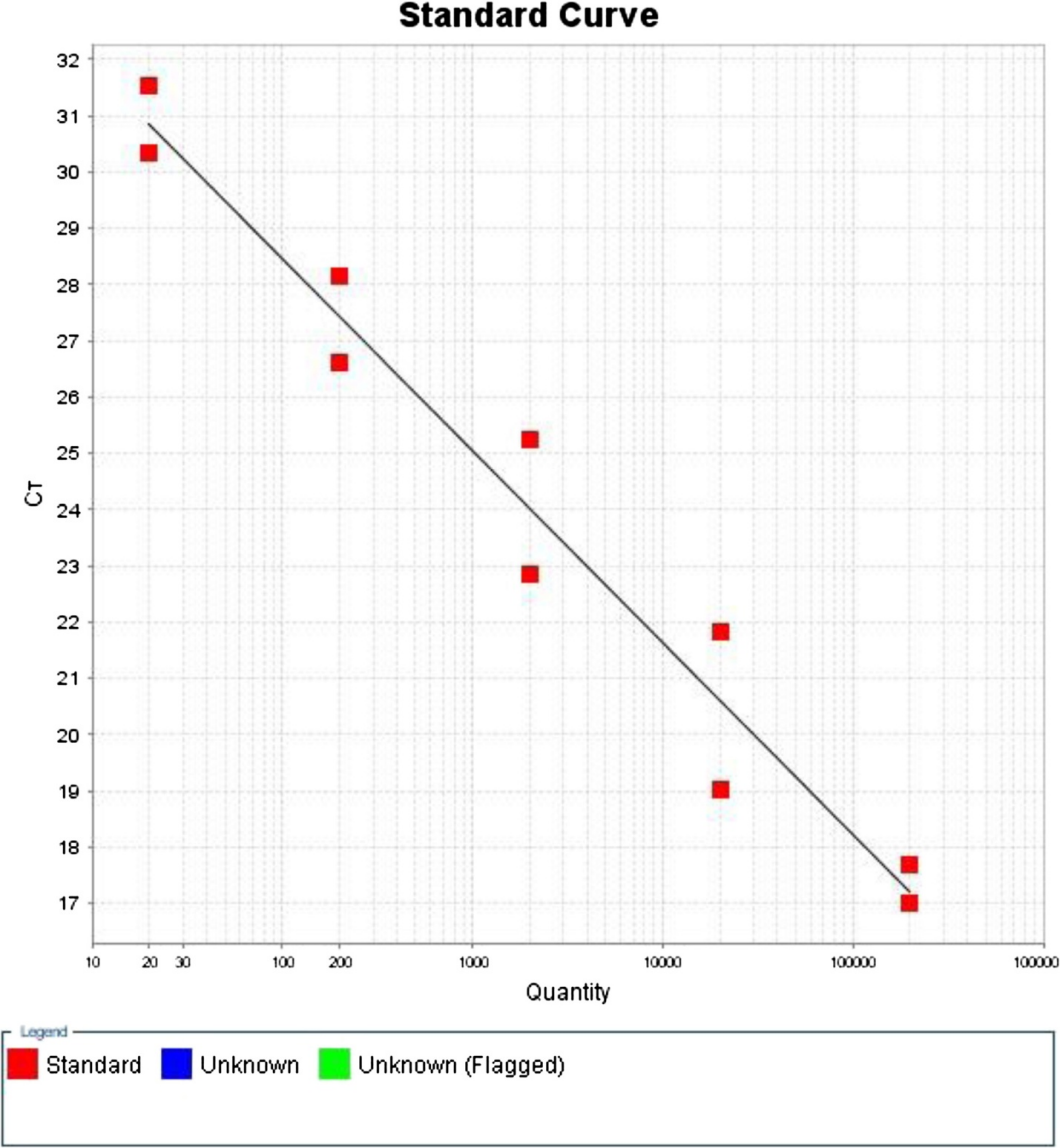
Standard curve obtained by 10-fold dilution series of the positive control

Positive and negative controls were included in each assay. The positive control was purchased from the Vircell Company. The concentration of HHV-6 control DNA was 10^6^ copies/μL. The assay was validated by using a 10-fold dilution series of the positive control. The detection limit for the Real time PCR was 20 copies of DNA /μL (Fig. 1).

The test was carried out with Qiagen Probe Real time PCR kit (Germany). The thermocycling procedure consisted of denaturation at 95 °C for three minutes, 45 cycles of denaturation at 95 °C for 15 s, annealing at 55 °C for 30 s and extension at 60 °C for 30 s. The Applied Biosystems StepOne Plus machine was used for PCR amplification.

### Statistical analysis

Categorical variables are reported through frequencies (percentages) and continuous variables are presented as mean (SD). The Independent *t* test was used to compare the mean of continuous variables between the cases and controls. The distribution of qualitative variables was compared through Chi-squared and Fisher’s exact tests between the groups. Statistical analysis was performed using SPSS 18.0 software (SPSS Inc., Chicago, IL, USA). P-values less than 0.05 were considered statistically significant.

## Results

### Type specific Real time PCR

A total of 120 patients (60 SC and 60 BD) and 75 HCs were included in this study. To investigate the prevalence of HHV-6A/B, a method of type specific Real time PCR with two different probes was used to test the presence of HHV-6A/B in DNA extracted from the PBMCs. Only one case of HHV-6B out of 120 (0.8 %) SC and BD patients and two cases of HHV-6A (2.7 %) in 75 HC subjects were detected in total samples. One hundred copies of viral DNA were detected in the positive samples. No association was found between HHV-6 infection and SC and BD.

### Demographic characteristics of the study population

In SC patients there was no difference in season of birth (25 % was born in each season) but in BD patients 35 % were born in winter, 25 % in spring, 25 % in summer and 15 % in autumn. The single BD patient positive for HHV-6B was born in spring with a family history of BD. Ethnicity, country of origin and socioeconomic status, were similar across the groups. Other demographic characteristics are shown in Table 2.

**Table 2:** Demographic characteristics of study population

	Cases			Controls (*N* = 75)	P-value
	SC	BD	Total		
	(*N* = 60)	(*N* = 60)	(*N* = 120)		
Age					
Mean (SD)	35.95 (9.99)	35.87 (11.58)	35.91 (10.77)	35.59 (10.74)	0.839^a^
Sex					
Male (%)	46 (76.7 %)	32 (53.3 %)	78 (65.0 %)	48 (64.0 %)	0.887^b^
Female (%)	14 (23.3 %)	28 (46.7 %)	42 (35.0 %)	27 (36.0 %)	
Marital status					
Single (%)	41 (68.3 %)	31 (52.5 %)	72 (60.5 %)	35 (48.5 %)	0.090^c^
Married (%)	17 (28.3 %)	27 (45.8 %)	44 (37.0 %)	37 (51.4 %)	
Divorced (%)	2 (3.4 %)	1 (1.7 %)	3 (2.5 %)	0 (0.0 %)	
Family history					
Yes (%)	18 (30.0 %)	26 (43.3 %)	44 (36.7 %)	-	-
No (%)	42 (70.0 %)	34 (56.7 %)	76 (63.3 %)		
Hospital Admission history					
Yes (%)	58 (96.7 %)	56 (93.4 %)	114 (95 %)	-	-
No (%)	2 (3.3 %)	4 (6.6 %)	6 (5 %)		
Smoking					
Yes (%)	31 (61.7 %)	14 (23.3 %)	45 (37.5 %)	8 (10.7 %)	<0.001^b^
No (%)	29 (48.3 %)	46 (76.7 %)	75 (62.5 %)	67 (89.3 %)	

*SC* Schizophrenia, *BD* Bipolar disorder, *SD* Standard deviation

^a^Significances of all cases vs. controls are based on *t*-test

^b^Significances of all cases vs. controls are based on Chi- square test

^c^Significances of all cases vs. controls are based on Fisher-exact test

## Discussion

The detection of HHV-6 DNA in PBMCs and plasma has been proposed as a marker of latent and active viral replications, respectively [16]. As the detection of HHV-6 in plasma might be negative while positive in PBMCs [17], this study was performed for detection of HHV-6A/B in PBMCs of SC and BD patients with a type specific Real time PCR and compared the results with HCs to find the latent infections. However, the detection level of HHV-6 in PBMCs was very low, which limited the ability of this study to investigate differences between the controls and BD or SC patients in this study population.

Schizophrenia and BD are polygenic mental disorders with major contribution of developmental and environmental factors [18]. Although several lines of evidence indicates that infectious agents may play a role in the pathogenesis of SC and BD, there is no strong research data showing a definite link between viral agents and these neuropsychiatric disorders.

Herpes viruses have been generally considered as potential contributing factors in the development of psychiatric disorders. Human herpes virus 6 is a member of the family of *herpesviridae* and the subfamily of *betaherpesvirinae* and is associated with widespread infections in humans [19]. Problems related to HHV-6 infections are ranged from simple infections to neuropsychiatric complications, though there are diagnostic challenges and therapeutic approaches to be addressed [20]. While two distinct species of HHV-6 have been identified, exanthema subitum as the first point of recognizing HHV-6 infection mostly occurs with HHV-6B [21]. The human herpes virus-6A is more prevalent than HHV-6B in patients with neurological diseases [22]. In a study by Nitsche et al. [23] on paired samples of peripheral blood leukocytes (PBL) and plasma of 25 patients with bone marrow transplant and 30 HCs, the load of HHV-6A DNA in plasma was higher than HHV-6B, but in HCs no HHV-6A/B DNA was detected in PBL and plasma.

The neuroinvasiveness of HHV-6 is contingent to the fact that its DNA is frequently found in specimens from different regions of the human brain [24–29]. Until now the hypothesis on the association between HHV-6 and psychotic diseases remains interesting, though unproven. It was stated that the HHV-6 latent protein, SITH-1, may contribute in the increased risk of mood disorders in chronic fatigue syndrome and psychosis (Kobayashi N, Shimada K, Kuratsune H, Kondo K: Identification of novel HHV-6 neurovirulent latent protein that causes mood disorders in CFS, psychosis and HHV-6 en cephalopathy, Unpublished data). In contrast Fukuda et al. [30] found no increase in HHV-6 antibody in paired sera of eight patients with the acute exacerbation of SC.

This case–control study analyzing 120 patients with SC and BD and 75 HCs found no evidence for the association of HHV-6 with SC and BD using type-specific Real time PCR analysis. This method provides a rapid and type-specific assay for detecting and genotyping HHV-6 in clinical specimens. According to this assay, HHV-6B was detected in a 27 years old female with BD and HHV-6A was found in two HCs without a history of any psychiatric disorders. Overall this study was not able to detect any statistical significant differences in the prevalence of HHV-6 DNA in patients with SC and BD in comparison with HCs. Therefore, it remains controversial whether HHV-6 infection is associated with SC and BD in humans. Hence, further studies on different samples, specially CSF and brain biopsies using other advanced diagnostic tools are recommended to elucidate the presence of HHV-6 in these patients.

Many studies have shown that individuals with SC have 5–15 % excess of winter and spring births [31–33] which is not observed in SC patients of the present study. Similar to our findings, some studies on patients with BD have shown a winter-spring seasonal birth excess [1] which might be the result of higher viral exposures during these seasons.

Our current study has a number of limitations to be under consideration in future studies. First, we have not been able to differentiate and exclude the carriers of ci-HHV-6. Second, PCR analysis of specimens from brain biopsy and CSF for HHV6A/B should be more specific and sensitive for diagnosing HHV-6 related to psychotic disorders. While brain biopsy has many ethical limitations in living individuals, with the current state of knowledge we have no convicting evidence to collect CSF samples from these individuals. Third, there are not adequate numbers of studies about HHV-6 in psychotic patients to verify the results of our findings. Forth, with a moderate sample size we initially designed this study to use a sensitive method for detection of HHV-6 DNA in PBMCs to uncover latent infections in patients and HCs. Next, we planned to continue this study for the detection of viral DNA in plasma and compare the viral load in patients and HCs to find active infection and its possible relationship with these diseases. However because of the low detection rate in PBMCs we decided to stop working on plasma with the current sample size.

## Conclusion

In conclusion, there was a lack of association of HHV-6 infection with SC and BD in the present study and huge challenges are remained to elucidate the etiology of SC and BD in humans.

Meanwhile, as there is not a single type-specific assay to detect HHV-6A/B, we recommend this Real time PCR based assay for rapid and simultaneous detection and typing of the HHV-6A/B viruses.

## Abbreviations

BD: Bipolar disorder
BHQ-1: Black hole quencher 1
CNS: Central nervous system
CSF: Cerebrospinal fluid
DNA: Deoxyribonucleic acid
DSM-IV: Diagnostic and Statistical Manual of Mental Disorders, Fourth Edition
FAM: 6-carboxyfluorescein
JOE: 6-carboxy-4,5-dichloro-2,7-dimethyoxyflurorescein
HC: Healthy control
HHV-6: Human herpes virus-6
PBMC: Peripheral blood mononuclear cell
PCR: Polymerase chain reaction
SC: schizophrenia
SCID-I: Structured Clinical Interview for DSM-IV Axis I Disorders
TAMRA: Tetramethylrhodamine
